# Sugiol Suppresses the Proliferation of Human U87 Glioma Cells via Induction of Apoptosis and Cell Cycle Arrest

**DOI:** 10.1155/2022/7658899

**Published:** 2022-05-29

**Authors:** Saif A. Alharthy, Shams Tabrez, Ahmed A. Mirza, Torki A. Zughaibi, Chelapram K. Firoz, Mycal Dutta

**Affiliations:** ^1^Department of Medical Laboratory Sciences, Faculty of Applied Medical Sciences, King Abdulaziz University, Jeddah, Saudi Arabia; ^2^King Fahd Medical Research Center, King Abdulaziz University, Jeddah, Saudi Arabia; ^3^Department of Medical Laboratory Technology, MIMS College of Allied Health Sciences, ASTER MIMS Academy, Kerala University of Health Sciences, Kerala, India; ^4^Department of Pharmacy, BGC Trust University Bangladesh, Chittagong 4381, Bangladesh

## Abstract

The diterpenoid, sugiol, has been reported to exert anticancer effects against a number of human cancers. However, the anticancer effects of sugiol have not been evaluated against the human glioma cells. The present study was designed to examine the effects of sugiol on the proliferation of human U87 glioma cells. The results showed that sugiol significantly (*P* < 0.05) suppressed the viability of the U87 cells in a concentration dependent manner and exhibited an IC_50_ value of 15 *μ*M. On the other hand, the growth inhibitory effects of sugiol were minimal on the normal human astrocytes. Acridine orange and ethidium bromide staining (AO/EB) staining revealed that sugiol induces apoptosis which was further confirmed by Western blot analysis, wherein upregulation of Bax and downregulation of Bcl-2 were observed in U87 cells. Flow cytometry showed that sugiol causes cell cycle arrest at the *G*_0_/*G*_1_ stage. The relative percentage of G1 phase was found to be increased from 26.58% at 0 *μ*M to 70.96% at 30 *μ*M sugiol. Taken together, the results suggest sugiol inhibits the growth of glioma cells and may prove to be a lead molecule in the management of human glioma.

## 1. Introduction

Gliomas are the form of tumors of the brain and spinal cord emerging from the glial cells which act as nerve supporters [1–5]. More than 30% of tumors of the central nervous system are the gliomas which account for up to 80% of all the malignant tumors of the human brain [6]. Glioblastoma is the most common malignant subtype of glioma, which frequently affects the adult human brain with an annual frequency of around 1 per 33000 people [7]. The glioblastomas are highly lethal with a 5-year survival rate as low as 6% for elder people [8]. The gliomas are currently being treated by the combinatorial application of surgical resection and chemo/radiotherapy. These treatment procedures are seen with limited clinical success, and patients affected with advanced stages of glioma die within 16 months [9]. Therefore, it becomes necessary to search for more effective treatment measures against glioma. Herein, the anticancer effects of sugiol were investigated against the glioma cells, in vitro. Sugiol is a diterpenoid type of plant-based natural product which is often isolated from the bark of *Calocedrus formosana* Florin (Cupressaceae) through ethanol extraction [10]. Sugiol has been shown to exhibit considerable antioxidant and anti-inflammatory properties. Besides, the antiproliferative effects are also known for sugiol against a number of human cancer cell lines. It has shown to inhibit the in vitro growth of prostate cancer cells through STAT3 targeting [11]. Also, the sugiol treatment induced apoptosis and cell cycle arrest and blockage of the RAF/MEK/ERK signaling pathway in ovarian cancer cells [12]. However, the anticancer effects of sugiol and its mechanism of action have not been evaluated till date against the glioma cancer cells. In the present study, the glioma cell line U87 was administered with varied concentrations of sugiol (98% pure by HPLC), and its cytotoxic effects were investigated. Sugiol was shown to exhibit an IC_50_ value of 15 *μ*M against the glioma cells, whereas the inhibitory effects were considerably less prominent against the human astrocytes. The anticancer effects of sugiol against glioma cells were exerted through induction of apoptosis. The glioma cells were also shown to be induced for cell cycle arrest at the *G*_0_/*G*_1_ phase by sugiol in vitro treatment. The results thus highlight the potency of sugiol to serve as lead molecule in developing chemotherapeutic agents against glioma, and its potency might be enhanced through semisynthetic chemistry approaches.

## 2. Materials and Methods

### 2.1. In Vitro Cell Culturing

The glioma cell line (U87) and the normal human astrocytes were procured from American Type Culture Collection (ATCC, USA). Dulbecco's modified Eagle medium (DMEM, PAN Biotech) was used for the cell culturing at 37°C with 5% CO_2_ saturation. The culture medium was supplemented with 10% fetal bovine serum (FBS).

### 2.2. (4,5-Dimethylthiazol-2-yl)-2,5-diphenyl Tetrazolium (MTT) Assay

2.5 × 10^4^ U87 cancer cells or astrocytes were inoculated per well of 96-well plates. The wells were added with variable doses of sugiol. The untreated samples were used as a control for the proliferation study. After 24 h growth at 37°C, 10 *μ*L MTT reagent (2.5 mg/mL) (Sigma-Aldrich, Germany) was added to each well and incubation was extended for 2 h again, after which the cells were harvested and resuspended in dimethyl sulfoxide (DMSO). Finally, absorbance for each sample was recorded using the microplate reader at 570 nm.

### 2.3. AO/EB Staining Apoptosis Assay

For studying the cancer cells apoptosis, the U87 cells were cultured for 24 h without or with varying concentrations of sugiol in 12-well plate. Centrifugation was performed for harvesting the cells which were ethanol fixed. The cells were then stained with a solution of acridine orange and ethidium bromide (AO/EB). Poststaining, the cells were examined for nuclear fluorescence under the fluorescent microscope.

### 2.4. Western Blotting

RIPA lysis and extraction buffer (Thermo Fisher Scientific, USA) was used for digesting the sugiol treated or untreated U87 cancer cells to isolate the total proteins. After quantification using Bradford assay, 40 *μ*g of total proteins from each sample was resolved for band separation on SDS-PAGE gels. The separated proteins were transferred electrophoretically to nitrocellulose membranes. The membranes were then incubated with primary antibodies (Bax (sc-7480, Santa Cruz, CA, USA), Bcl-2 (sc-23960, Santa Cruz, CA, USA), and actin (sc-58673, Santa Cruz, CA, USA)) at 4°C overnight (dilution for all antibodies were kept 1 : 1000). The proteins were then incubated with horseradish peroxidase-conjugated anti-rabbit secondary antibody (sc-2357-CM; Santa Cruz, CA, USA). The enhanced chemiluminescent substrate was finally used for protein band detection. Human actin protein served as an internal control in protein loading.

### 2.5. Flow Cytometry

The U87 cancer cells were treated with varying concentrations of sugiol (7.5 *μ*M, 15 *μ*M, or 30 *μ*M) for 24 h at 37°C, while the untreated cells were used as negative control. Afterwards, the cells were harvested, PBS washed, methanol fixed, and then treated with propidium iodide (PI). For the cell cycle study, the treated cancer cells were analyzed using flow cytometry.

### 2.6. Statistical Analysis

The statistical analysis was performed using GraphPad Prism 5.0 software for determining the significance level between the treatment groups. The same was achieved through Student's *t*-test or one-way ANOVA. *P* values <0.05 represented the significant level of statistical difference. Results were given as mean ± standard deviation. Three replicates were used for each experiment.

## 3. Results

### 3.1. Selective Cytotoxic Action of Sugiol against the Glioma Cells

**Figure 1:**
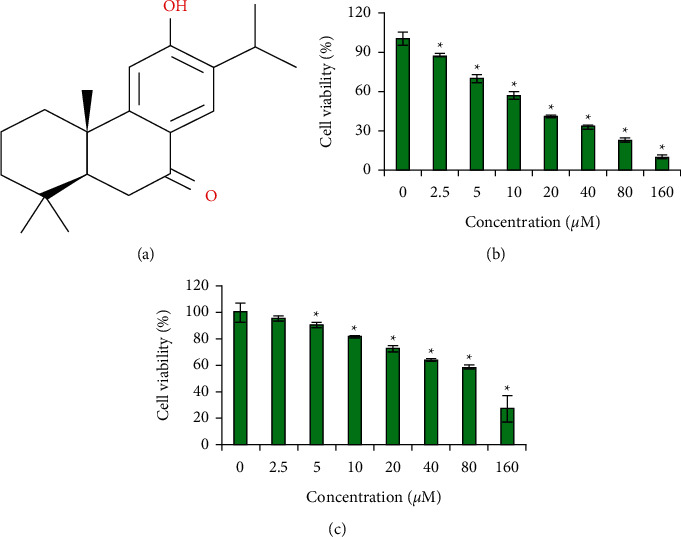
Diterpenoid sugiol selectively inhibits the glioma cell growth. (a) Chemical structure of sugiol. (b) Assessment of viability of glioma cancer cells administered with variable sugiol concentrations. (c) Analysis of viability of normal human glial cells treated with different concentrations of sugiol (^*∗*^*P* < 0.05).

Figure 1(a) shows the chemical structure of sugiol. The U87 glioma cells were treated with different concentrations of sugiol (0–160 *μ*M). Sugiol was found to significantly (*P* < 0.05) inhibit the U87 cell growth in a concentration-dependent manner with an IC50 value of 15 *μ*M (Figure 1(b)). Similar treatment doses of sugiol when applied against normal human astrocytes were less effective to determine the astrocyte cell growth in vitro, and IC50 (90 *μ*M) was much higher than as observed against the glioma cells. The results indicate the selective cytotoxic property of sugiol against the glioma cells.

**Figure 2:**
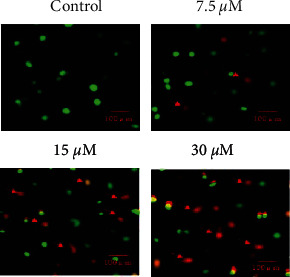
Sugiol induces apoptosis in human glioma cells. AO/EB staining of glioma cells differentially treated with or without sugiol.

**Figure 3:**
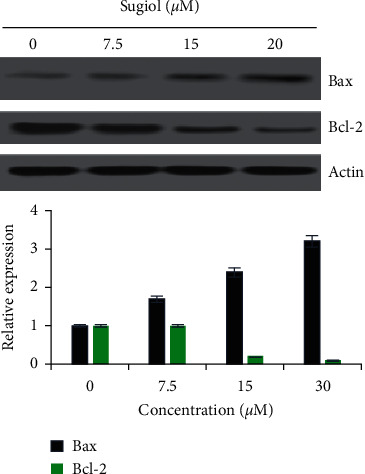
Sugiol modulates Bax/Bcl-2 signaling ratio in human glioma cells. Analysis of expression levels of Bax and Bcl-2 from glioma cells administered with different sugiol concentrations with reference to untreated cancer cells. G0/G1 phase cell cycle arrest of glioma cells by sugiol.

AO/EB staining of U87 cancer cells treated with different concentrations of sugiol with reference to untreated cancer cells underwent considerable nuclear deformation which was much prominent when higher treatment doses were used (Figure 2). The red and orange color cells increased remarkably suggestive of apoptosis. Western blotting of Bax and Bcl-2 proteins indicated with the increasing concentration of sugiol, the Bax protein expression was increased, while the expression of Bcl-2 protein showed reverse trend (Figure 3). Both the results support that apoptosis is induced in glioma cells when treated with sugiol that led to decline in cell growth.

**Figure 4:**
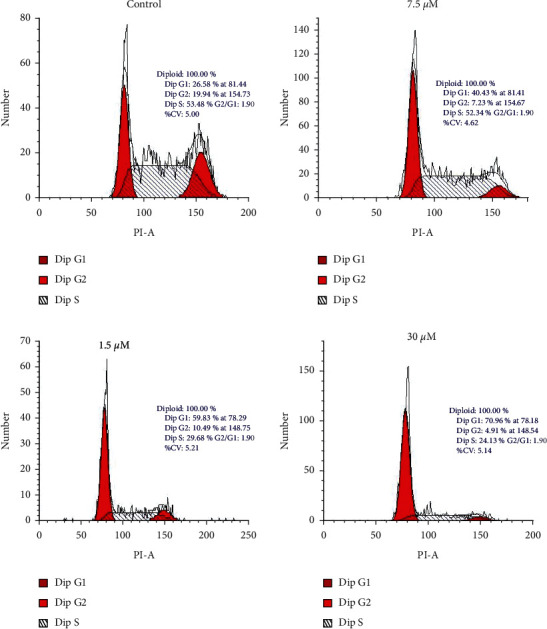
Sugiol induces G_0_/G_1_ phase cell cycle arrest in human glioma cells. Cell cycle phase distribution of glioma cells administered with variable sugiol doses.

To find whether sugiol treatment altered the progression of glioma cell mitosis, the sugiol treated or untreated U87 cancer cells were analyzed for cell cycle phase distribution with the help of flow cytometry. The sugiol treatment significantly increased the relative percentage of G0/G1 phase cells in a dose-dependent manner (Figure 4). The relative percentage of G1 phase in U87 cancer cells increased from 26.58% at 0 *μ*M to 70.96% at 30 *μ*M sugiol concentrations. Sugiol is thus active in inducing G_0_/G_1_ phase arrest of glioma cell mitosis to inhibit the cell growth, in vitro.

## 4. Discussion

Although gliomas are comparatively rare, its malignant forms, like glioblastoma, are highly lethal and in advanced stages, and the survival period is less than one and a half years [13]. The prognosis of malignant gliomas is very difficult adding to the disease aggressiveness [14]. The combinatorial treatment procedure of surgery followed by radio/chemotherapy is currently in use against glioma. The radio/chemotherapies aimed to prevent the tumor recurrence at original or distant sites after surgical resection. The application of radiations is seen with negative effects such as necrosis of neural tissues [15]. The chemotherapy in many cases is opposed by the attainment of resistance to drug molecules by the cancer cells [16]. Hence, researchers are continuously focusing on formulating better treatment methods against glioma, and for this purpose, and continuous efforts are underway. In the present study, the anticancer effects of sugiol were worked out against the glioma cells. The results indicated that sugiol exhibits selective cytotoxic property against the glioma cells, while the effects were abysmally less prominent against the normal cell population. The finding is highly encouraging for the possible utility of sugiol in glioma chemotherapy. The selective growth inhibitory action of sugiol is also known for other cancer cell types including ovarian and prostate cancer cells [12, 13]. Sugiol has previously been reported to exhibit proapoptotic effects [17]. The results of the present study also support the same. The induction of apoptosis was shown to be the possible mechanism mediating the antiproliferative effects of sugiol against glioma cells as suggested by nuclear deformation and modulation of Bax/Bcl-2 signaling ratio. The efficacy of sugiol against the glioma cells was besides evident from the fact that glioma cells were inducted with cell cycle arrest at G_0_/G_1_ phase cell cycle arrest when treated with sugiol in vitro. Similar mechanism of action of the anticancer role against the pancreatic cancer cells has already been deduced by a previous research study [18]. G_0_ to G_1_ phase transition is critical for mitotic entry of eukaryotic cells and sugiol treatment and tends to prevent the mitotic entry of glioma cells to reduce their proliferation in vitro [19, 20]. Summing up, sugiol exhibits anticancer effects against human cells through induction of apoptosis and G_0_/G_1_ cell cycle arrest and may prove beneficial in the management of human glioma. However, more studies are required, especially in vivo in nature to further validate the outcomes of this study.

## 5. Conclusion

The results of the present study are supportive of anticancer activity of sugiol against U87 cells. The induction of apoptosis, modulation of Bax/Bcl-2 signaling ratio, and cell cycle arrest at G0/G1 phase of cell cycle seems to be the possible mechanism of action of sugiol in glioma cells. Collectively, the results suggest the anticancer potential of sugiol that could be utilized in the management of human glioma and its potency might be enhanced through semisynthetic chemistry approaches.

## Data Availability

The data used to support this study are included within the article.

## References

[1] Lapointe S., Perry A., Butowski N. A. (2018). Primary brain tumours in adults. *The Lancet*.

[2] Gowd V., Ahmad A., Tarique M. (2022). Advancement of cancer immunotherapy using nanoparticles-based nanomedicine. *Seminars in Cancer Biology*.

[3] Zughaibi T. A., Mirza A. A., Suhail M. (2022). Evaluation of anticancer potential of biogenic copper oxide nanoparticles (CuO NPs) against breast cancer. *Journal of Nanomaterials*.

[4] Abuzenadah A. M., Al-Sayes F., Mahafujul Alam S. S. (2022). Identification of potential poly (ADP-ribose) polymerase-1 inhibitors derived from *Rauwolfia serpentina*: possible implication in cancer therapy. *Evidence-Based Complementary and Alternative Medicine*.

[5] Abuzenadah A. M., Al-Sayes F., Mahafujul Alam S. S. (2022). Elucidating anti-angiogenic potential of *Rauwolfia serpentina*: VEGFR-2 targeting based molecular docking study. *Evidence-based Complementary and Alternative Medicine*.

[6] Rehan M., Mahmoud M. M., Tabrez S., Hassan H. M. A., Ashraf G. M. (2020). Exploring flavonoids for potential inhibitors of a cancer signaling protein PI3K*γ* kinase using computational methods. *Anticancer Research*.

[7] Herholz K. (2017). Brain tumors: an update on clinical PET research in gliomas. *InSeminars in nuclear medicine*.

[8] Reni M., Mazza E., Zanon S., Gatta G., Vecht C. J. (2017). Central nervous system gliomas. *Critical Reviews In Oncology-Hematology*.

[9] Tamimi A. F., Juweid M. (2017). *Epidemiology and Outcome of Glioblastoma*.

[10] Ostrom Q. T., Cote D. J., Ascha M., Kruchko C., Barnholtz-Sloan J. S. (2018). Adult glioma incidence and survival by race or ethnicity in the United States from 2000 to 2014. *JAMA Oncology*.

[11] Chao K. P., Hua K. F., Hsu H. Y., Su Y. C., Chang S. T. (2005). Anti-inflammatory activity of sugiol, a diterpene isolated from Calocedrus formosana bark. *Planta Medica*.

[12] Jung S. N., Shin D. S., Kim H. N. (2015). Sugiol inhibits STAT3 activity via regulation of transketolase and ROS-mediated ERK activation in DU145 prostate carcinoma cells. *Biochemical Pharmacology*.

[13] Wang Y., Shi L. Y., Qi W. H., Yang J., Qi Y. (2020). Anticancer activity of sugiol against ovarian cancer cell line SKOV3 involves mitochondrial apoptosis, cell cycle arrest and blocking of the RAF/MEK/ERK signalling pathway. *Archives of Medical Science*.

[14] Gately L., McLachlan S. A., Philip J., Ruben J., Dowling A. (2018). Long-term survivors of glioblastoma: a closer look. *Journal of Neuro-Oncology*.

[15] Jabir N. R., Anwar K., Firoz C. K., Oves M., Kamal M. A., Tabrez S. (2018). An overview on the current status of cancer nanomedicines. *Current Medical Research and Opinion*.

[16] Tsai S. R., Hamblin M. R. (2017). Biological effects and medical applications of infrared radiation. *Journal of Photochemistry and Photobiology B: Biology*.

[17] Wang X., Li X., Dai X. (2018). Coaxial extrusion bioprinted shell-core hydrogel microfibers mimic glioma microenvironment and enhance the drug resistance of cancer cells. *Colloids and Surfaces B: Biointerfaces*.

[18] Tayarani-Najaran Z., Mousavi S. H., Tajfard F. (2013). Cytotoxic and apoptogenic properties of three isolated diterpenoids from Salvia chorassanica through bioassay-guided fractionation. *Food and Chemical Toxicology*.

[19] Hao C., Zhang X., Zhang H. (2018). Sugiol (127horbar;hydroxyabieta-8,11,13-trien-7-one) targets human pancreatic carcinoma cells (Mia-PaCa2) by inducing apoptosis, G2/M cell cycle arrest, ROS production and inhibition of cancer cell migration. *Journal of BUON*.

[20] Champion L., Linder M. I., Kutay U. (2017). Cellular reorganization during mitotic entry. *Trends in Cell Biology*.

